# Shear Bond Strength of Clear Aligner Attachment Using 4-META/MMA-TBB Resin Cement on Glazed Monolithic Zirconia

**DOI:** 10.3390/polym16141988

**Published:** 2024-07-11

**Authors:** Kasidit Nitasnoraset, Apiwat Riddhabhaya, Chidchanok Sessirisombat, Hitoshi Hotokezaka, Noriaki Yoshida, Irin Sirisoontorn

**Affiliations:** 1 Department of Clinical Dentistry, Walailak University International College of Dentistry (WUICD), 87 Ranong 2 Road, Dusit, Bangkok 10300, Thailand; 2 Department of Orthodontics and Dentofacial Orthopedics, Nagasaki University Graduate School of Biomedical Sciences, 1-7-1 Sakamoto, Nagasaki 852-8588, Japan

**Keywords:** clear aligner attachment, monolithic zirconia, mode of failure, shear bond strength

## Abstract

Increasing demand for adult orthodontic treatment using clear aligners has highlighted challenges in bonding clear aligner attachments to various restorations. Specifically, the bond strength of clear aligner attachments to glazed monolithic zirconia has not been extensively studied. This study aims to compare the shear bond strength (SBS) and mode of failure (MOF) of conventional bonding methods versus Superbond C&B (4-META/MMA-TBB resin cement) for clear aligner attachments on glazed monolithic zirconia. Fifty sintered and glazed zirconia samples were divided into five groups and attached with clear aligner attachments: Si (silane), B (bonding agent), SiB (bonding agent and silane), SU (Superbond C&B), and SiSU (silane and Superbond C&B). SBS and MOF of these samples were analyzed. Results indicated a significant difference in bond strength among the groups. SiSU exhibited the highest bond strength, followed by SU, while B had the lowest bond strength. SEM analysis revealed that SiSU and SU predominantly exhibited mixed failure, indicating high bond strength without affecting the glazed layers of the zirconia. In contrast, B exhibited only adhesive failure at the interface, resulting in insufficient bond strength for effective orthodontic treatment. In conclusion, using 4-META/MMA-TBB resin cement provides high bond strength for clear aligner attachments on glazed zirconia with minimal material damage during debonding.

## 1. Introduction

Traditionally, ceramics were developed to satisfy the dental industry’s demand for aesthetically pleasing, natural-looking restorations. However, these conventional ceramics exhibit significant drawbacks, including low tensile strength, susceptibility to cracking and wear, fragility, compromised fracture resistance, marginal integrity, and reparability challenges [1]. Since the 1990s, zirconia (zirconium dioxide, ZrO_2_) emerged as a dental material, favored for its role as optimum properties for dental material, including superior toughness (9–10 MPam^1/2^), higher strength when compared to traditional ceramics (900–1200 MPa), fatigue resistance, excellent wear properties, and biocompatibility [2]. These attributes make zirconia the strongest and hardest dental ceramic available [3]. Dental zirconia is commonly a modified yttria (Y_2_O_3_) tetragonal zirconia polycrystal (Y-TZP). With the addition of nanostructure modification, yttria stabilizes the crystal structure transformation during high-temperature firing, thereby enhancing the physical properties of zirconia, including strength and translucency [4].

The development of digital technology, particularly CAD/CAM systems, has revolutionized dental restorations by enabling precise and efficient digital construction [5], contributing to the rising popularity of monolithic zirconia options for prosthetic restorations. Yttria-stabilized tetragonal zirconia polycrystal (Y-TZP) is favored as a core material due to its enhanced fracture resistance from transformation toughening. However, the high fracture risk of porcelain veneer layering, likely due to mismatched thermal expansion coefficients between zirconia and porcelain, remains a significant concern [6]. To mitigate these failures, the glazing surface of monolithic zirconia-based dental prostheses has been developed. This technique involves applying a thin glass coating to zirconia surfaces or heating for a glossy finish, improving the aesthetics and clinical efficacy of zirconia restorations [7]. Additionally, glazed monolithic zirconia is less abrasive to opposing teeth compared to its polished counterpart [8].

Recently, there has been a notable rise in adult orthodontic patients, with a significant interest (approximately 45%) observed in the middle-aged demographic [9]. Frequently, adult patients begin orthodontic treatment with missing teeth, and they may also have prosthesis restorations or other dental conditions that increase the complexity of treatment. With the popularity of clear aligners, the demand for adult orthodontic treatment has increased significantly [10], which are preferred for their superior hygiene, comfort, aesthetics, fewer required visits, and shorter treatment duration compared to traditional fixed appliances [11].

Kesling introduced the approach of employing clear overlay orthodontic appliances to facilitate tooth movement in 1945 and suggested the progressive alignment of teeth through the utilization of a series of thermoplastic tooth positioners [12]. In 1997, Align Technology (Invisalign™; Santa Clara, CA, USA) advanced this concept by incorporating digital technology, including 3D graphic imaging and CAD/CAM modeling, to develop clear aligner treatments. This technology allows for the fabrication of a series of precise aligners to provide comprehensive orthodontic treatment. Clear aligners have been praised for being a safe, esthetic, and convenient orthodontic option for patients [13]. The Invisalign system offers a specialized technique for correcting malocclusions as prescribed by orthodontists. Recent enhancements aimed at improving effectiveness and widening the scope of treatable cases have been categorized under modified aligner shapes, power ridge features, and active attachment surfaces, which enhance biomechanical control and tooth movement predictability [14].

Aligner attachments consist of composite resin bonded to the tooth surface, which plays a crucial role as auxiliary devices in enhancing the effectiveness of various clear aligners by transmitting forces from the aligner to target pressure on a specific tooth. Modern resin composites have advanced significantly in terms of formulation and performance, incorporating several key components for improved clinical outcomes. These include high-strength resin matrix (such as Bis-GMA and UDMA), enhanced inorganic filler particles (including nano-sized fillers for superior polishability and mechanical strength), advanced silane coupling agents for better bonding between resin and fillers, and optimized initiator systems (such as camphorquinone combined with co-initiators) for efficient polymerization. During the curing process, these monomers undergo polymerization, forming a cross-linked polymer network. This polymer network provides the structural framework that binds the inorganic filler particles and other components, giving the composite resin its strength, durability, and ease of use for a wide range of restorative applications [15]. These attachments come in various shapes, aiding in improved retention and precise control over specific tooth movements whenever necessary. Comprising composite resin securely bonded to the tooth surface, attachment loss can occur due to bond failure or patient neglect, potentially leading to significant clinical complications that might extend treatment duration, necessitate additional visits, and affect treatment outcomes [16]. A recent study demonstrated that operator-related is one of the factors that can cause attachment loss [17], especially with orthodontic treatment in patients with fixed prostheses who face challenges in effectively bonding brackets and clear aligner attachments. With heightened esthetic expectations and the growing prevalence of monolithic zirconia, orthodontists are more likely to encounter patients with both cosmetic preferences and existing zirconia restorations. Bonding composite resin to this existing zirconia frequently causes confrontation complications [18].

Systematic reviews show the protocol for bonding to porcelain surfaces involves applying a silane coupling agent after treating the surface with 9.6% hydrofluoric acid. This process forms a strong covalent bond between porcelain and composite resin [19]. Additionally, many studies demonstrate methods to create strong bond strength between porcelain and composite resin using resin cement, such as 4-META/MMA-TBB resin cement (Superbond C&B) [20,21].

**Figure 1:**
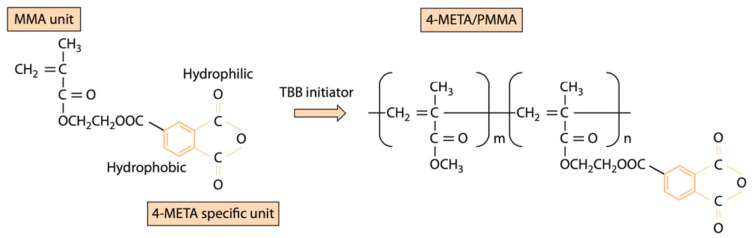
The chemical structure of 4META/MMA molecule with TBB initiator, which undergoes polymerization and forms 4META/MMA-TBB resin cement [23].

**Figure 2:**
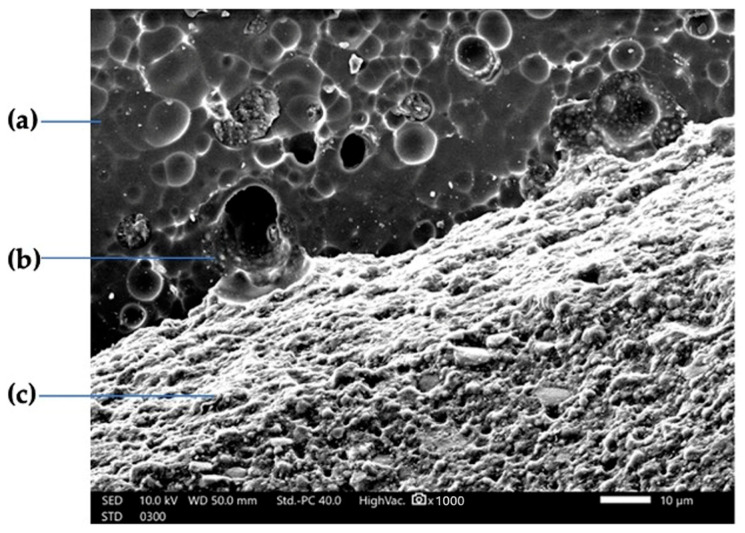
SEM micrograph with 1000× magnification of the zirconia surface after being treated with HF and superbond C&B; (**a**) zirconia surface; (**b**) resin-impregnated layer; (**c**) Superbond C&B.

Super-bond C&B (Sun Medical Co Ltd., Moriyama, Japan) is a 4-methacryloloxyethyl trimellitate anhydride (4-META)/methyl methacrylate (MMA)-tri-n-butyl borane (TBB) resin. The main ingredients of monomer, co-polymer, diffusion promoter, and catalyst S are MMA, 4-META, and TBB, respectively [22]. The 4-META/MMA-TBB resin cement, used extensively in dental applications for its superior adhesive properties, is a composite material formed from the polymerization of three key components: 4-META, MMA, and TBB initiator. The 4-META molecule, which integrates a methacrylate group with a trimellitate anhydride group, enhances the adhesive bond to dentin, while MMA serves as a monomer that polymerizes to form a durable polymer network. TBB functions as an initiator, triggering the polymerization process (Figure 1). This combination results in a resin cement that is highly effective in creating strong, lasting bonds in dental restorative procedures [23]. The formation of a hybrid layer [24] is facilitated by the diffusion of the 4-META monomer and the TBB catalyst onto the structure’s surface (Figure 2). Super-Bond C&B structure exhibits high plastic deformation and easily changes shape. This resilience provides a significant advantage over traditional adhesive cements [25]. Due to its slight flexibility after curing, Superbond forms a stronger and more durable bond that can withstand higher levels of pressure from biting forces.

For orthodontic appliances, effective tooth movement requires the bond strength between the teeth and appliances to exceed necessary forces. An appropriate range of 5.9–7.9 MPa is recommended for this purpose [26]. Research on the bonding properties of glazed monolithic zirconia has primarily focused on metal brackets, with results indicating excessively high bond strength values (>13 MPa) [27]. Recent research has demonstrated the impact of various types of cement used between metal orthodontic appliances on glazed monolithic zirconia [28]. Additionally, using Superbond C&B as cement to resin composite shows high bond strength [29]. Nevertheless, the investigation to improve the bond strength between clear aligner attachment and glazed monolithic zirconia has not been studied so far.

The objective of this research was to compare the shear bond strength (SBS) and failure mode (MOF) of conventional approaches with the utilization of 4-META/MMA-TBB resin cement for the bonding of attachments to glazed monolithic zirconia.

## 2. Materials and Methods

### 2.1. Sample Size Calculation

The pilot study utilized G Power software (version 3.1) to determine the sample size, indicating a requirement for a group of 7 samples based on an effect size of 0.8933026 and an actual power of 0.9619761. To account for potential errors, the sample sizes were subsequently increased to 10 specimens per group.

### 2.2. Sample Preparation

**Figure 3:**
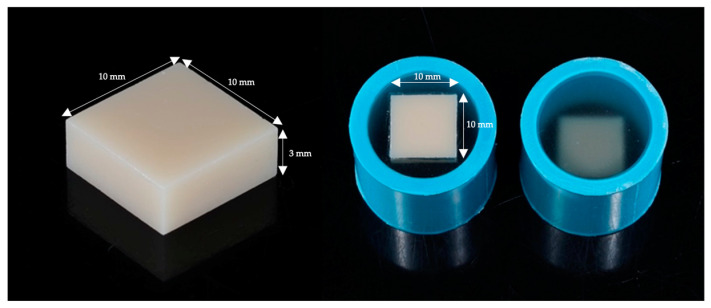
Zirconia blocks were sintered, glazed, and embedded in a polyvinylchloride tube fixed with pouring resin.

In this research, disk-shaped zirconia (Cercon^®^, Dentsply Sirona™, Charlotte, NC, USA) were milled and segmented among 50 specimens by CAD/CAM technology, each measuring 10 × 10 × 3 mm^3^. These zirconia blocks completed sintering (The inLab Profire sintering furnace, Dentsply Sirona™) and glazing (Ducera^®^ Liquid STAIN, Dentsply Sirona™) processes according to the steps specified by the manufacturer. Subsequently, all sintered and glazed zirconia blocks were embedded into polyvinylchloride tubes fixed with self-cure acrylic resin using Shofu pouring resin (Tokyo, Japan) [28,30] (Figure 3).

Each sample will have its surface treated by etching with 9.6% hydrofluoric acid gel (Porcelain Etch, Ultradent™, South Jordan, UT, USA) for 1 min. This will be followed by a thorough rinse with water for 30 s to remove any residual acid, and then the samples will be air-dried [19]. Sequentially, the samples were evenly distributed into five groups, differentiated by their bonding methods: Si: attachment utilizing silane (3M™ Silane Coupling Agent, 3M ESPE, St. Paul, MN, USA); B: attachment using a bonding agent (3M Transbond™ XT Primer, 3M ESPE, St. Paul, MN, USA); SiB: attachment employing both bonding agent and silane; SU: attachment with Superbond C&B resin cement (Sun Medical Co., Ltd., Moriyama, Japan); SiSU: attachment combining silane and Superbond C&B resin cement.

In the B and SiB groups, the bonding process involves applying a thin layer of bonding agent to the designated bonding area on the zirconia surface, followed by air-drying for approximately 10 s. The bonding agent is then lightly cured using an Ortholux luminous curing light (3M Unitek, 3M ESPE, St. Paul, MN, USA) for 20 s. To ensure the light source’s output power density remains between 350–470 mW/cm^2^, a 3 Light Checker (3M Health Care, Tokyo, Japan) is used. In the Si, SiB (prior to bonding agent application), and SiSU groups, the surface of zirconia was chemically treated using a silane coupling agent. A thin layer of the silane coupling agent was applied to the clean, dry zirconia surface and allowed to air-dry for 60 s, following the manufacturer’s recommendations.

For the composite attachment, an Invisalign clear aligner template (Invisalign system, Align technology, Santa Clara, CA, USA) was used to create composite attachments featuring a trapezoid-shaped cutout. The cutout, tailored to the attachment’s specifications, had parallel sides measuring 1.5 mm and 2 mm, a height of 4 mm, and a thickness of 2 mm. Prior to bonding, the template appliance was cleaned with 75% ethyl alcohol and then air-dried [17]. After surface pretreatment, the composite resin (Z350, 3M ESPE, St. Paul, MN, USA) was placed into the Invisalign clear aligner template [31]. The template was then positioned on the zirconia surface and compressed with a weight of 150 g [21] for the B, Si, and SiB groups. The remaining composite resin was meticulously eliminated following exposure to visible light utilizing a light-emitting diode light source (Ortholux luminous curing light; 3M Unitek, 3M ESPE, St. Paul, MN, USA) for a duration of 40 s.

**Figure 4:**
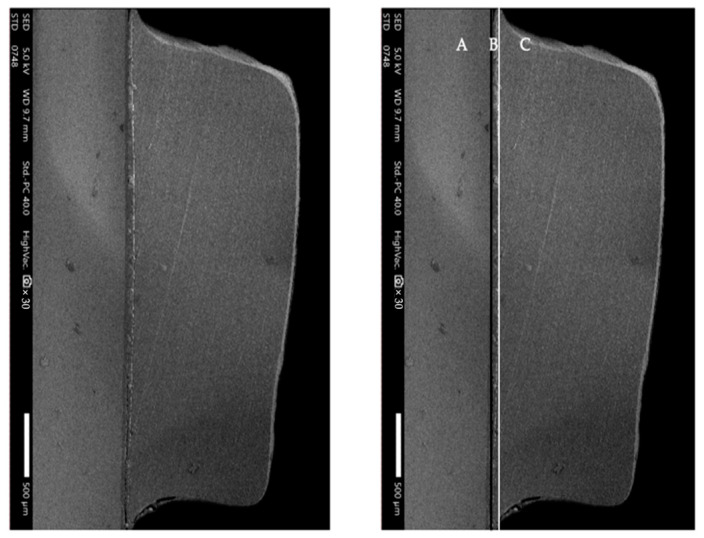
A representative specimen of the glazed monolithic zirconia attached with clear aligner attachment with cross-sectional SEM image (original magnification 30×); A, zirconia layer; B, adhesive layer; C, clear aligner attachment layer; black line, zirconia-adhesive interface; white line, adhesive-base of attachment interface.

**Figure 5:**
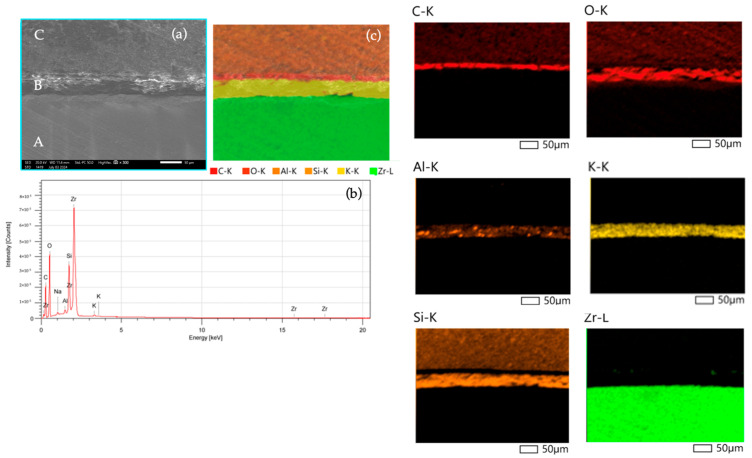
The SEM-EDS (Energy Dispersive X-ray Spectroscopy) analysis image confirms the interface between the zirconia surface and composite resin attachment. In the SEM image, area A is identified as the zirconia layer, area B as the adhesive layer, and area C as the clear aligner attachment layer. (**a**) SEM image; (**b**) EDX analysis; (**c**) Elemental mapping analysis. Elemental mapping shows a thin, red-colored layer near the top with a high presence of carbon, indicating resin cement and resin composite. This area also shows a prominent presence of oxygen, suggesting an oxide layer. An orange-colored layer signifies a significant silicon presence in the composite resin, with scattered regions indicating minor aluminum content. The yellow-colored map shows potassium distributed along a specific horizontal layer, indicating its presence in the 4-META/MMA-TBB resin cement included as part of the catalyst system. Potassium is typically used to enhance adhesive properties and promote polymerization. The green-colored area reveals a substantial zirconium presence, confirming the zirconia layer predominantly in the lower part of the interface.

In the SU and SiSU groups, composite attachments were prepared by placing resin composite into the Invisalign template, pressing it with 150 g on a flat surface, and then light curing for 40 s. The Superbond C&B was prepared according to the instructions and applied to the base of the cured resin composite attachment. This attachment was then placed on the pretreated zirconia surface (Figure 4). Following proper positioning, the samples were pressed with 150 g force using a loading device, consistent with the protocol of a previous study [21]. The Superbond C&B resin cement was allowed to cure autonomously for 6 min, following the manufacturer’s guidelines. Additionally, the SEM-EDS (Energy Dispersive X-ray Spectroscopy) analysis image confirms the interface between the zirconia surface and composite resin attachment (Figure 5).

### 2.3. Data Collection

#### 2.3.1. Surface Characterization (Scanning Electron Microscopy Observation)

The surface of the sample was gold-coated and meticulously polished (Jeol-smart coater-DII-29030SCTR, Jeol LTD, Tokyo, Japan). A scanning electron microscope (SEM; Jeol-JSM-IT700HR, Jeol LTD, Tokyo, Japan) was used to study the failure mode of the attachment and zirconia surface morphologies.

#### 2.3.2. Shear Bond Strength

**Figure 6:**
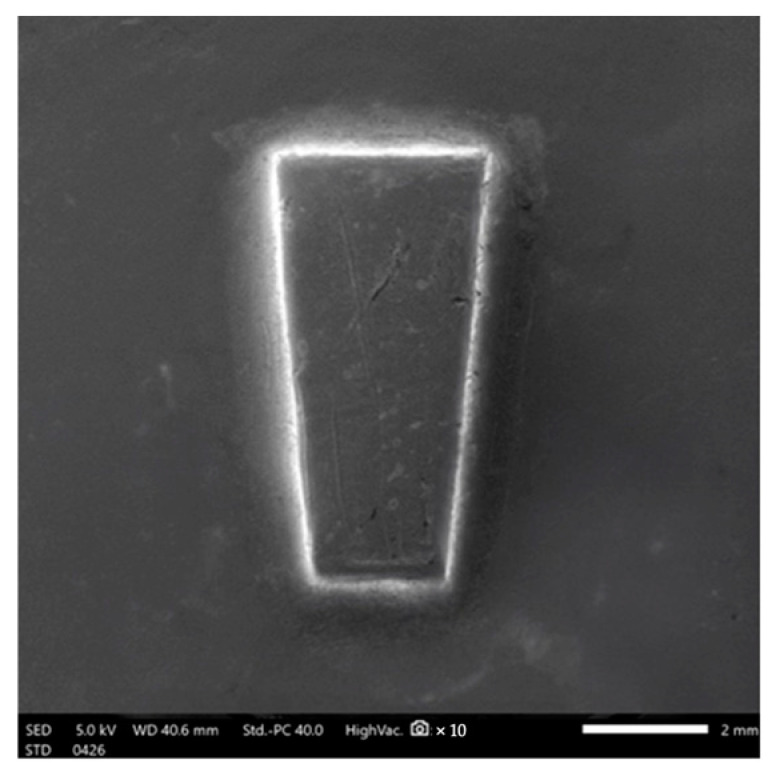
The areas of the attachment bases were calculated using image analysis software (ImageJ software version 0.5.7, NIH, Bethesda, MD, USA) as 7.0253 mm^2^.

The shear bond strength was evaluated with universal testing equipment (Model 5566; Instron^®^ Co., Norwood, MA, USA), adhering to ISO/TS 11405:2015 (E) [32]; Dental materials — Testing of adhesion to tooth structure. International Organization for Standardization: Geneva, Switzerland, 2015 [28]. Prior to testing, samples were immersed in distilled water at 37 °C for 24 h. During the test, the zirconia surface was aligned parallel to the applied force, generating shear force at the attachment–zirconia interface by transferring a vertical load to the attachment base. The shear load was assessed using a crosshead speed of 0.75 mm/min. The resulting data were standardized to MPa by dividing them by the area of the attachment base. The attachment bases’ areas were determined using image analysis tools (ImageJ program version 0.5.7, NIH, Bethesda, MD, USA) and found to be 7.0253 mm^2^ (Figure 6).

#### 2.3.3. Mode of Failure

Following the shear load test, the locations of failure were assessed by examining the images of the debonded attachment surfaces obtained with a scanning electron microscope and light microscope. Using the Adhesive Remnant Index (ARI) to quantify the residual adhesive on zirconia surfaces after removing attachments, a digital transparent grid was superimposed on the images. This method quantifies the quantity of adhesive that remains on the zirconia surface by calculating it as a proportion of the entire attachment area. The ARI is utilized to identify and classify the bonding failure locations and types between the interface of the adhesive, the zirconia surface, and the attachment. Through this scoring system, the method not only measures the leftover adhesive post-debonding but also helps in elucidating the bonding failure’s nature, distinguishing between adhesive (at the bonding interface), cohesive (within the adhesive or material), or mixed types of failures [30].

**Figure 7:**
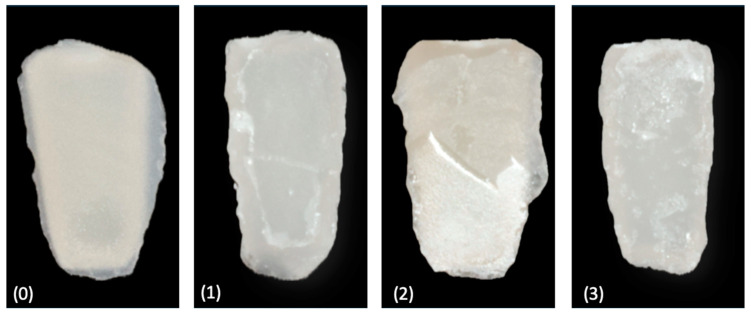
The adhesive remnant index (ARI) encompasses the following values: (**0**) indicates the absence of adhesive on the zirconia surface; (**1**) indicates less than 50% adhesive remnants on the zirconia surface; (**2**) indicates more than 50% adhesive remnants on the zirconia surface; and (**3**) indicates the total amount of adhesive remnants on the zirconia surface.

The ARI scoring for zirconia surfaces typically follows the traditional 0 to 3 scale; 0 means no adhesive remains on the zirconia surface, indicating that all the adhesive has been removed or stayed with the attachment; 1 means less than half of the adhesive remains on the zirconia, showing that most of the adhesive was removed with the attachment; 2 means more than half of the adhesive remains on the zirconia, revealing less than half of the adhesive on the attachment; 3 means all of the adhesive remains on the zirconia, presenting no adhesive on the attachment (Figure 7).

### 2.4. Data Analysis Strategies

In order to assess the attributes of the specimens, descriptive statistics were utilized. The Kolmogorov–Smirnov test was utilized to determine whether the distribution of the data was normal. Using a one-way ANOVA, differences in shear bond strengths between distinct groups were evaluated. Whenever non-homogeneous variances were present, Tamhane’s T2 test was applied to analyze discrepancies among the means of several groups. The statistical analyses were conducted using SPSS 22.0 for MacOS IBM (SPSS Inc., Chicago, IL, USA), with a significance threshold set at α < 0.05.

## 3. Results

### 3.1. Shear Bond Strength

**Figure 8:**
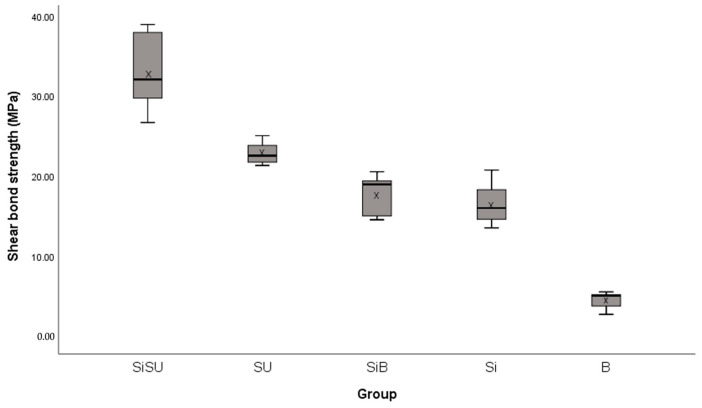
Box plots represent the shear bond strength values of clear aligner attachments bonded to glazed monolithic zirconia surfaces; X indicates the mean SBS value of each group.

The descriptive statistics, as presented in Table 1 and Figure 8, refer to the SBS values (MPa) of all groups. ANOVA and Tamhane’s T2 for multiple comparison tests revealed that the mean SBS values of at least two groups differed significantly (F) (4, 45) = 150.214, *p* < 0.01). Table 1 and Table 2 reveal that the SiSU group exhibited considerably greater SBS values (33.25 ± 4.57 MPa) in comparison to the other groups. The SU group provided the second SBS value (22.88 ± 1.29 MPa). In addition, there was no statistical difference between the SiB (17.72 ± 2.46 MPa) and the Si group (16.58 ± 2.56 MPa). The B group provided the lowest SBS value (4.55 ± 0.99 MPa).

### 3.2. Mode of Failure

The ARI scores of the five groups differed significantly, as determined by the chi-square test (chi-square = 53.846, df = 8, *p* = 0.000). The ARI ratings of 1 (40%), 2 (50%), and 3 (10%) for the SiSU group suggested a substantial quantity of adhesive remaining on the surfaces of the zirconia. The ARI scores for the SU, SiB, and Si groups were mostly 1 and 2, with excellent results of 10/10 for each group, suggesting that the zirconia surfaces retained a negligible amount of adhesive. However, the ARI scores of the samples in group B were entirely zero (one hundred percent), indicating that the zirconia surface was devoid of any adhesive residue subsequent to the rupture of the attachment (Table 3).

## 4. Discussion

### 4.1. Shear Bond Strength

The advent of high-strength zirconia ceramics, combined with CAD/CAM technology, has significantly advanced all-ceramic restorations, enhancing their design and application. However, zirconia’s chemical inertness presents challenges for achieving chemical adhesion with traditional bonding techniques effective for other ceramics containing glass, such as acid etching and silanization. This study emphasizes the need for detailed research on adhesive bonding procedures to improve the durability and efficacy of zirconia-based restorations.

According to Reynolds (1975) [26], a minimum shear bond strength (SBS) range of 6–8 MPa is necessary to withstand typical orthodontic forces. Our results show that the SiSU, SU, SiB, and Si groups bonded to glazed monolithic zirconia achieved the required and higher SBS values (33.25 ± 4.57 MPa, 22.88 ± 1.29 MPa, 17.72 ± 2.46 MPa, and 16.58 ± 2.56 MPa, respectively). Conversely, the B group exhibited lower SBS values (4.55 ± 0.99 MPa) than what is considered adequate for conventional orthodontic tooth movement. This finding highlights the crucial importance of 4-META/MMA-TBB resin cement and silanization in improving the bond strength of the glazed surfaces of monolithic zirconia.

The study specifically compared the effects of using Superbond C&B and silane alone on the shear bond strength (SBS) of clear aligner attachments to glazed monolithic zirconia. The results showed that Superbond C&B alone achieved an SBS of 22.88 ± 1.29 MPa, which is substantially higher than the minimum requirement for orthodontic forces. This underscores the significant bonding capability of Superbond C&B due to its unique chemical composition and properties, which allow for effective adhesion even without additional agents.

The crucial role of 4-META/MMA-TBB resin cement (Superbond C&B) in improving bond strength between resin composite was highlighted [33]. In this resin composite system, bis-GMA provides a rigid backbone due to its aromatic bisphenol A core, while its glycidyl methacrylate groups participate in polymerization, creating a strong, cross-linked polymer network. 4-META, with its methacrylate group and trimellitate anhydride functionality. When bis-GMA and 4-META are combined, the methacrylate groups from both components participate in the formation of covalent bonds, resulting in a highly cross-linked polymer network. This network integrates the structural rigidity of bis-GMA with the adhesive properties of 4-META, providing strong, durable bond strength between 4META/MMA-TBB resin cement and resin composite attachment. Additionally, Superbond C&B’s ability to form a hybrid layer, also known as a resin-impregnated layer, contributes to its superior bond strength. This layer is a result of the resin penetrating the microstructural features of the zirconia surface and polymerizing within these spaces, creating a mechanically interlocked interface. This hybrid layer significantly enhances the bond’s mechanical stability and resistance to stress. According to the small molecular weight of MMA [24], it allows for high diffusivity, meaning it can penetrate deeper into the micro-gaps and irregularities on the zirconia surface. This deep penetration ensures a more comprehensive and secure bond, which is critical for maintaining bond strength over time and under various conditions.

In contrast, the use of silane alone (Si group) resulted in an SBS of 16.58 ± 2.56 MPa. This value is above the minimum SBS requirement. Silane coupling agents enhance bond strength by forming covalent bonds (siloxane networks) [34] with inorganic filler particles and improving the wettability [35] of the composite substrate surface, thus promoting the penetration of the bonding agent. The combined use of silane produced superior results with the excellent diffusivity and hybridization properties of Superbond C&B and the covalent bonding and wettability enhancement provided by silane. Previous studies also support the effectiveness of Superbond C&B, showing high microtensile bond strength between zirconia ceramics and resin composite, with values ranging from 43.3 to 53.9 N/mm^2^ [36]. The other study [37] discovered that Superbond provides great bond strength (9.7 ± 3.1 MPa) on ceramic surfaces. In addition, the inclusion of silica coating and silanization resulted in significantly increased bond strengths, with values of 20.2 ± 3.7 MPa.

In the study of bonding properties between composite resin and hybrid ceramics like glazed zirconia, mechanical and chemical pretreatments are recommended, including hydrofluoric acid (HF) etching and sandblasting. While grinding with diamond burs can lead to high stress and reduced strength in zirconia, sandblasting [38,39] with aluminum oxide particles (110 μm at 0.2 MPa for 20 s) is more effective, although it may not create sufficient undercuts for strong bonding after thermocycling [40]. HF etching, particularly with 9.6% HF, produces prominent etching patterns and enhances bond strength even after thermocycling. Studies indicate that silica-coated surfaces treated with HF exhibit increased surface roughness and improved bonding strength when using silane coupling agents [41]. Other studies reported that treated silica-coated surface materials such as lithium disilicate and glazed monolithic zirconia using 9.6 hydrofluoric acid produced greater surface roughness (Ra) [42] and showed a significant increase in the enhancing bond strength of resin cement via silane coupling agents [43]. Therefore, in this study, all specimens treated with HF showed that the SBS values for each group increased when Superbond C&B and silane were introduced, as follows: The mean value of the SU group was 22.88 ± 1.29 MPa, whereas the SiSU group had a mean value of 33.25 ± 4.57 MPa. The mean value of the B group was 4.55 ± 0.99 MPa, whereas the SiB group had a mean value of 17.72 ± 2.46 MPa. The utilization of silane alone or the combination of silane with a bonding agent elevated the shear bond strength (SBS) value. Conversely, the B group, which did not use silane or 4-META/MMA-TBB resin cement, exhibited lower SBS values (4.55 ± 0.99 MPa), inadequate for conventional orthodontic tooth movement.

### 4.2. Mode of Failure

**Figure 9:**
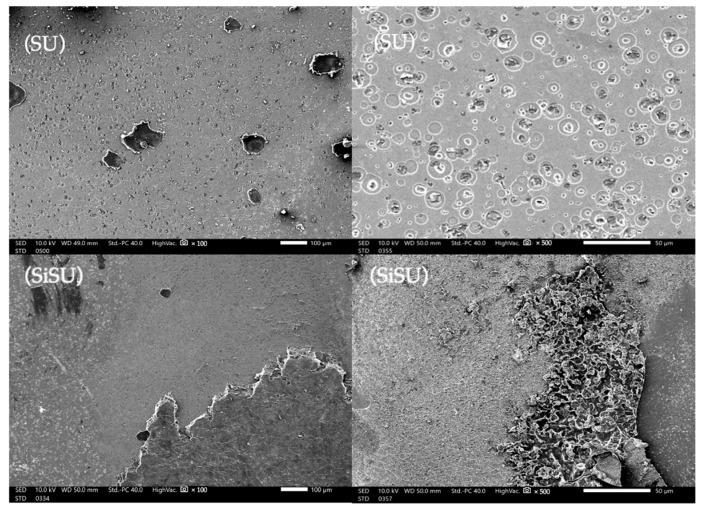
SEM examination at 100× and 500× magnification showed that both the zirconia sites in the SU and SiSU groups had a combination of adhesive and cohesive failure on the debonded surfaces. There were no instances of adhesive failure reported at the interfaces between the ceramic–cement or resin composite–cement.

The mode of failure (MOF) can be broadly classified into three main types: cohesive failure, adhesive (interfacial) failure, and mixed-mode failure. Each of these failure modes has distinct characteristics and implications for the performance and reliability of the material system. First, cohesive failure occurs within a single material when the internal forces causing the material to break are stronger than the forces holding it together. This type of failure is indicative of the material’s inherent strength and is often preferred in bonded joints because it demonstrates that the bond is stronger than the material itself. Second, adhesive (interfacial) failure occurs specifically at the interface between two materials, where the bond strength is weaker than the strength of either material [44]. The third one, mixed failure, represents a combination of cohesive and adhesive failures occurring both within the materials themselves and at their interface. It suggests that the bond between materials and the materials themselves have similar strength levels, leading to a failure that involves both materials detaching from each other and breaking apart internally [45]. To prevent damage to zirconia surfaces during attachment debonding, ensuring minimal adhesive residue is essential. A low ARI means reduced cohesive failures and decreased adhesive remnants on the surfaces. The SEM analysis at 100× and 500× magnification, as shown in Figure 9, revealed that both the zirconia and attachment sites in the SiSU and SU groups exhibited a mix of adhesive and cohesive failure on the debond surfaces. No adhesive failure was observed along the ceramic–cement or resin composite–cement interfaces.

**Figure 10:**
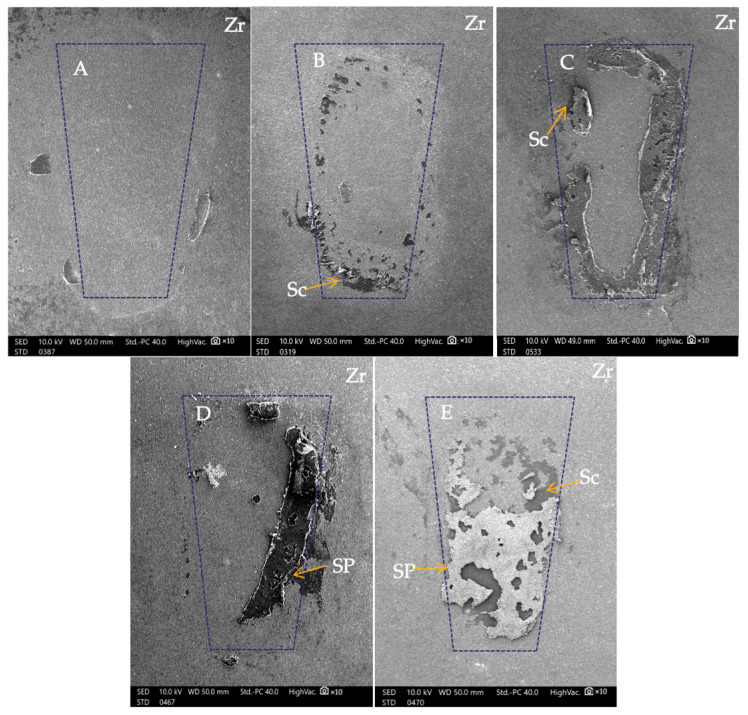
SEM micrograph 10× magnification of surface characteristics on the zirconia surface after shear bond strength test. The dotted loops represent 7 mm^2^ of composite attachment base, Zr: Zirconia, SP: Superbond C&B, Sc: Silane coupling agent. (**A**) Adhesive failure occurred at the interface between the zirconia surface and composite attachment base. (**B**) Mixed failure includes a small part of silane on the zirconia surface. (**C**) Mixed failure includes the part of silane–zirconia interface failure as well as a part of cohesive failure in composite attachment. (**D**) Mixed failure includes the part of zirconia–adhesive interface failure with a little adhesive area between adhesive and attachment. (**E**) Mixed failure includes zirconia–adhesive interface failure and cohesive failure in resin.

Mixed failure, usually associated with high bond strength, occurred predominantly within the adhesive for the SiSu, SU, SiB, and Si groups (Figure 10). Particularly, these failures did not affect the glazed layers of the zirconia. Nevertheless, a single specimen from the SiSU group had an ARI score of 3, suggesting the presence of a large adhesive residue on the surface of the zirconia material. Cohesive failures within the ceramic material would happen when a force greater than 13 MPa. After debonding, the restoration surfaces may be at risk of damage due to the highly strong bond strength. This may be the only drawback of the debonding procedure [46]. In this study, no fractures in the glazing surface were found in any group. This might be because Superbond C&B contains a methacrylate in the MMA-based monomer, enhancing its flexural strength. Additionally, Superbond C&B was too elastic to fracture. Its high plasticity is likely due to the high proportion of the TBB catalyst in the cured cement, a unique characteristic of 4-META/MMA-TBB resin cement.

In the B group, only adhesive failure was observed at the interface between adhesive and zirconia (Figure 10). There is a correlation with 100% adhesive failure found in this group with 100 percent ARI of 0, which means the weakest point was at the connection between the material and the resin cement. There is no adhesive retaining on the zirconia surface as a result of unnecessary damage to the material surface; however, the SBS values are insufficient for achieving the minimal threshold for tooth movement. Lacking a silane coupling agent during the bonding process, the adhesive was incapable of adhering to the silica oxide present on the surfaces of the glazed monolithic zirconia.

This study explores the impact of clear aligner attachments on glazed monolithic zirconia, focusing on bonding materials from a single company. While providing insights into bonding strength and failure modes, the study’s findings are limited by the specific brand used, potentially restricting their applicability to other materials and bonding techniques. To advance clinical practice, further investigation should encompass a broader array of bonding materials and methods, aiming to enhance understanding of material interactions in dental restorations. Additionally, exploring the effects of strain rate on bonding strength, investigating fatigue behavior, and conducting tensile bond strength testing will significantly enhance the durability and effectiveness of dental treatments.

## 5. Conclusions

- The highest shear bond strength (SBS) is achieved when a clear aligner attachment is bonded to a glazed monolithic zirconia surface using 4-META/MMA-TBB resin cement (Superbond C&B) in combination with a silane coupling agent.
- The use of 4-META/MMA-TBB resin cement (Superbond C&B) alone for bonding clear aligner attachments to glazed monolithic zirconia surfaces presents a groundbreaking innovation by eliminating the need for additional silane coupling agents while achieving high shear bond strength (SBS) without damaging the surface. This method streamlines clinical procedures, reduces potential errors, and maintains the integrity of the zirconia surface, offering a simplified and effective bonding solution.
- Traditional methods requiring multiple bonding agents and silane treatments are more time-consuming and prone to procedural errors. Therefore, the exclusive use of Superbond C&B is a significant advancement in dental restorative practices, providing both durability and stability for attachments, and the use of bonding agents alone is not recommended.

## Figures and Tables

**Table 1:** Shear bond strength (SBS) data of each group (MPa).

Group	n	Mean ± SD	Max	Min
SiSU	10	33.25 ± 4.57 ^a^	38.92	26.69
SU	10	22.88 ± 1.29 ^b^	25.04	21.32
SiB	10	17.72 ± 2.46 ^c^	20.53	14.54
Si	10	16.58 ± 2.56 ^c^	20.75	13.52
B	10	4.55 ± 0.99 ^d^	5.53	2.73

The one-way ANOVA findings showed substantial differences in the mean shear bond strength across the groups (*p* < 0.001). The Tamhane post hoc test demonstrated that there were no significant differences in the mean SBS values with the same superscripted letters (*p* > 0.05). Si: attachment utilizing silane; B: attachment using a bonding agent; SiB: attachment employing both bonding agent and silane; SU: attachment with Superbond C&B resin cement; SiSU: attachment combining silane and Superbond C&B resin cement. SD represents the standard deviation, Min stands for the least value, and Max represents the highest value.

**Table 2:** The mean differences in the shear bond strength values (MPa) for each group.

Group	SiSU	SU	SiB	Si	B
SiSU	-	10.37 *	15.53 *	16.67 *	28.7 *
SU		-	5.16 *	6.30 *	18.32 *
SiB			-	1.14	13.16 *
Si				-	12.02 *
B					-

* At *p* < 0.05, the Tamhane post hoc test indicated that the mean difference is statistically significant.

**Table 3:** The distribution of adhesive remnant index (ARI) for each group.

Group	n	ARI Score ^1^			
		0 (%)	1 (%)	2 (%)	3(%)
SiSU	10	0 (0)	4 (40)	5 (50)	1(10)
SU	10	0 (0)	6 (60)	4 (40)	0(0)
SiB	10	0 (0)	8 (80)	2 (20)	0(0)
Si	10	0 (0)	8 (80)	2 (20)	0(0)
B	10	10 (100)	0 (0)	0 (0)	0(0)

^1^ ARI, adhesive remnant index; 0 indicates the absence of adhesive on the zirconia surface; 1 indicates the presence of less than 50% adhesive; 2 indicates the presence of more than 50% adhesive; and 3 indicates the complete quantity of adhesive remaining on the zirconia surface.

## Data Availability

Data is contained within the article.
